# Complete Genome Sequence of Streptococcus salivarius DB-B5, a Novel Probiotic Candidate Isolated from the Supragingival Plaque of a Healthy Female Subject

**DOI:** 10.1128/MRA.00916-20

**Published:** 2020-10-01

**Authors:** Francisco R. Fields, Xuyao Li, William W. Navarre, Mizue Naito

**Affiliations:** a Dose Biosystems, Inc., Toronto, Ontario, Canada; b Department of Molecular Genetics, University of Toronto, Toronto, Ontario, Canada; University of Maryland School of Medicine

## Abstract

Streptococcus salivarius DB-B5 was isolated from the supragingival plaque of a healthy female subject. The complete 2.3-Mb genome consists of one circular chromosome, two circular plasmids (including a megaplasmid), and one linear phage-like episome. The genome possesses two separate loci encoding bacteriocins.

## ANNOUNCEMENT

Streptococcus salivarius is a normal commensal of the healthy human oral microbiome (1–3). Due to the species’ association with health and its ability to secrete antimicrobial compounds active against various pathogens, S. salivarius strains have been studied as potential probiotics for human health (4–7). To isolate a novel oral probiotic, supragingival plaque from a healthy Canadian female subject (African race) with no history of oral disease was plated on brain heart infusion (BHI) agar (Hardy Diagnostics) and incubated at 37°C for 48 h under both aerobic and anaerobic conditions. S. salivarius DB-B5 was identified among the facultative anaerobic colonies using colony PCR with the universal 16S rRNA primers 8F and 1492R (8). To further analyze DB-B5, whole-genome sequencing was performed.

S. salivarius DB-B5 was grown as described above, and genomic DNA was extracted using the DNeasy blood and tissue kit (Qiagen). Paired-end libraries (2 × 300 bp) were constructed using the Nextera XT library kit (Illumina) and sequenced on the MiSeq v3 platform (Illumina) at the Centre for the Analysis of Genome Evolution and Function at the University of Toronto. In parallel, DB-B5 genomic DNA was also extracted using the Wizard genomic DNA purification kit (Promega) and sent to Génome Québec for library preparation and PacBio sequencing. The PacBio library was prepared following the manufacturer’s instructions; DNA was sheared using g-TUBES (Covaris, Inc.), and SMRTbell templates were prepared using Express template preparation kit v2.0 reagents (Pacific Biosciences). The library was sequenced without size selection on the PacBio Sequel platform using the Sequel sequencing plate v3.0, single-molecule real-time (SMRT) cells (1M v3), and 10-h movies. Illumina reads were quality filtered using Trimmomatic (v0.38.0) (9). PacBio reads were checked for quality using FastQC (v0.72). A *de novo* hybrid assembly was performed using Unicycler (v0.4.8.0) with 1,722,228 Illumina paired-end reads and 629,563 PacBio long reads (*N*_50_, 139,339 bp), with a genome coverage of ∼1,000× (10). Unicycler automatically identified and trimmed overlapping ends, and circular elements were flipped and/or rotated to *dnaA*. The complete genome consists of one circular chromosome (2,143,863 bp, with a GC content of 40.2%), one megaplasmid named pIKMIN-B501 (138,497 bp, with a GC content of 35.6%), one small plasmid named pIKMIN-B503 (3,225 bp, with a GC content of 39.6%), and one linear phage-like episome named pIKMIN-B502 (57,714 bp, with a GC content of 39.1%). The linear ends of pIKMIN-B502 contain inverted terminal repeat sequences similar to those of other linear phages infecting Gram-positive cocci, confirming the likelihood that the linear ends are complete (11, 12). The genome was annotated by NCBI using the Prokaryotic Genome Annotation Pipeline (PGAP) (v4.11) (13). PGAP identified a total of 2,041 protein-coding genes, 18 complete rRNA genes, 4 noncoding RNA genes, and 68 tRNA genes. Default parameters were used for all software.

The S. salivarius DB-B5 genome was searched for bacteriocins using BAGEL4 (http://bagel4.molgenrug.nl) (14). The analysis revealed the presence of a thiazolyl peptide bacteriocin locus on the megaplasmid and a *blpU* bacteriocin locus on the chromosome. The genome sequence of S. salivarius DB-B5 will be useful for further understanding the mechanism of its probiotic properties.

### Data availability.

The complete genome sequences have been deposited in GenBank under the accession numbers CP054153 (chromosome), CP054154 (pIKMIN-B501), CP054155 (pIKMIN-B502), and CP054156 (pIKMIN-B503). The raw reads from both the PacBio and Illumina sequencing are available under the SRA BioProject accession number PRJNA635659.
